# Supplementary material for: Chlorophyll inversion in rice based on visible light images of different planting methods
Source: PLoS One. 2025 Mar 24;20(3):e0319657. doi: 10.1371/journal.pone.0319657 (PMC11932478; doi:10.1371/journal.pone.0319657)
Supplement: Attachment — Submitted filename: PONE-D-24-27430_reviewer- ghefra.pdf [file pone.0319657.s001.pdf]

PLOS ONE

Rice Chlorophyll Inversion Based on UAV Visible Light Imagery

--Manuscript Draft--

**Manuscript Number:**	PONE-D-24-27430
**Article Type:**	Research Article
**Full Title:**	Rice Chlorophyll Inversion Based on UAV Visible Light Imagery
**Short Title:**	Rice Chlorophyll Inversion
**Corresponding Author:**	jing he<br>Chengdu University of Technology<br>chengdu, CHINA
**Keywords:**	rice chlorophyll; unmanned aerial vehicle; visible light imagery; vegetation index.
**Abstract:**	As a key substance for crop photosynthesis, chlorophyll content is closely related to crop growth and health. Inversion of chlorophyll content using unmanned aerial vehicle (UAV) visible light images can provide a theoretical basis for crop growth monitoring and health diagnosis. Taking rice at the tasseling stage as the research object, we obtained UAV visible orthophotos of test areas A and B, constructed 14 vegetation indices and 15 texture features, and used the correlation coefficient method to comprehensively analyze the vegetation indices and texture features, and selected four vegetation indices and four texture features for test areas A and B as feature variables to participate in the three types of K-Nearest Neighbors (KNN), decision tree (DT) and AdaBoost models for the chlorophyll content inversion. In the KNN model, the inversion model built with BGRI as the independent variable in region A has the highest accuracy, with R2 of 0.666 and RSME of 0.79; the inversion model built with RGRI as the independent variable in region B has the highest accuracy, with R2 of 0.729 and RSME of 0.626. In the decision tree model, the inversion model built with B-variance as the independent variable in region A has the highest accuracy, with R2 of 0.840 and RSME of 0.464; the inversion model built with G-mean as the independent variable in region B has the highest accuracy, with R2 of 0.845 and RSME of 0.530. In the AdaBoost model, the inversion model built with R-skewness as the independent variable in region A has the highest accuracy, with R2 of 0.826 and RSME of 0.642; the inversion model established with g as the independent variable in area B had the highest accuracy, with R2 of 0.879 and RSME of 0.599. In the comprehensive analysis, the best inversion models in experimental areas A and B were B-variance-decision tree and g-AdaBoost, respectively, and their models were able to carry out the inversion of the chlorophyll content of rice quickly and accurately, which can provide a theoretical basis for the monitoring of the growth and health of the crop.
**Order of Authors:**	jing he
**Additional Information:**	
**Question**	**Response**
**Financial Disclosure**<br><br>Enter a financial disclosure statement that describes the sources of funding for the work included in this submission. Review the submission guidelines for detailed requirements. View published research articles from *PLOS ONE* for specific examples.<br><br>This statement is required for submission and **will appear in the published article** if	Yes

Please give some citations on background and methods

I think it would be better if you write at least last 5 years references

the submission is accepted. Please make sure it is accurate.<br><br>**Funded studies**<br>Enter a statement with the following details:<br>• Initials of the authors who received each award<br>• Grant numbers awarded to each author<br>• The full name of each funder<br>• URL of each funder website<br>• Did the sponsors or funders play any role in the study design, data collection and analysis, decision to publish, or preparation of the manuscript?<br><br>Did you receive funding for this work?	
Please add funding details.<br>as follow-up to "**Financial Disclosure**<br><br>Enter a financial disclosure statement that describes the sources of funding for the work included in this submission. Review the submission guidelines for detailed requirements. View published research articles from *PLOS ONE* for specific examples.<br><br>This statement is required for submission and **will appear in the published article** if the submission is accepted. Please make sure it is accurate.<br><br>**Funded studies**<br>Enter a statement with the following details:<br>• Initials of the authors who received each award<br>• Grant numbers awarded to each author<br>• The full name of each funder<br>• URL of each funder website<br>• Did the sponsors or funders play any role in the study design, data collection and analysis, decision to publish, or preparation of the manuscript?<br><br>Did you receive funding for this work?"	Chengdu City Technology Innovation R&D Project (2022-YF05-01090-SN)
Please select the country of your main	CHINA - CN

research funder (please select carefully as in some cases this is used in fee calculation).<br>as follow-up to "**Financial Disclosure**<br><br>Enter a financial disclosure statement that describes the sources of funding for the work included in this submission. Review the submission guidelines for detailed requirements. View published research articles from *PLOS ONE* for specific examples.<br><br>This statement is required for submission and **will appear in the published article** if the submission is accepted. Please make sure it is accurate.<br><br>**Funded studies**<br>Enter a statement with the following details:<br>• Initials of the authors who received each award<br>• Grant numbers awarded to each author<br>• The full name of each funder<br>• URL of each funder website<br>• Did the sponsors or funders play any role in the study design, data collection and analysis, decision to publish, or preparation of the manuscript?<br><br>Did you receive funding for this work?"	
**Competing Interests**<br><br>Use the instructions below to enter a competing interest statement for this submission. On behalf of all authors, disclose any competing interests that could be perceived to bias this work—acknowledging all financial support and any other relevant financial or non-financial competing interests.<br><br>This statement is required for submission and **will appear in the published article** if the submission is accepted. Please make sure it is accurate and that any funding sources listed in your Funding Information	The authors have declared that no competing interests exist.

later in the submission form are also declared in your Financial Disclosure statement.<br><br>View published research articles from *PLOS ONE* for specific examples.<br><br>**NO authors have competing interests**<br><br>Enter: *The authors have declared that no competing interests exist.*<br><br>**Authors with competing interests**<br><br>Enter competing interest details beginning with this statement:<br><br>*I have read the journal's policy and the authors of this manuscript have the following competing interests: [insert competing interests here]*<br><br>* typeset	
**Ethics Statement**<br><br>Enter an ethics statement for this submission. This statement is required if the study involved:<br><br>• Human participants<br>• Human specimens or tissue<br>• Vertebrate animals or cephalopods<br>• Vertebrate embryos or tissues<br>• Field research<br><br>Write "N/A" if the submission does not require an ethics statement.<br><br>General guidance is provided below. Consult the submission guidelines for detailed instructions. **Make sure that all information entered here is included in the Methods section of the manuscript.**	N/A

**Format for specific study types**<br><br>**Human Subject Research (involving human participants and/or tissue)**<br>• Give the name of the institutional review board or ethics committee that approved the study<br>• Include the approval number and/or a statement indicating approval of this research<br>• Indicate the form of consent obtained (written/oral) or the reason that consent was not obtained (e.g. the data were analyzed anonymously)<br><br>**Animal Research (involving vertebrate animals, embryos or tissues)**<br>• Provide the name of the Institutional Animal Care and Use Committee (IACUC) or other relevant ethics board that reviewed the study protocol, and indicate whether they approved this research or granted a formal waiver of ethical approval<br>• Include an approval number if one was obtained<br>• If the study involved *non-human primates*, add *additional details* about animal welfare and steps taken to ameliorate suffering<br>• If anesthesia, euthanasia, or any kind of animal sacrifice is part of the study, include briefly which substances and/or methods were applied<br><br>**Field Research**<br><br>Include the following details if this study involves the collection of plant, animal, or other materials from a natural setting:<br>• Field permit number<br>• Name of the institution or relevant body that granted permission	
**Data Availability**<br><br>Authors are required to make all data underlying the findings described fully available, without restriction, and from the time of publication. PLOS allows rare exceptions to address legal and ethical concerns. See the PLOS Data Policy and FAQ for detailed information.	Yes - all data are fully available without restriction

A Data Availability Statement describing where the data can be found is required at submission. Your answers to this question constitute the Data Availability Statement and **will be published in the article**, if accepted.<br><br>**Important:** Stating 'data available on request from the author' is not sufficient. If your data are only available upon request, select 'No' for the first question and explain your exceptional situation in the text box.<br><br>Do the authors confirm that all data underlying the findings described in their manuscript are fully available without restriction?	
**Describe where the data may be found in full sentences. If you are copying our sample text, replace any instances of XXX with the appropriate details.**<br><br>• If the data are **held or will be held in a public repository**, include URLs, accession numbers or DOIs. If this information will only be available after acceptance, indicate this by ticking the box below. For example: *All XXX files are available from the XXX database (accession number(s) XXX, XXX.*).<br>• If the data are all contained **within the manuscript and/or Supporting Information files**, enter the following: *All relevant data are within the manuscript and its Supporting Information files.*<br>• If neither of these applies but you are able to provide **details of access elsewhere**, with or without limitations, please do so. For example:<br><br>*Data cannot be shared publicly because of [XXX]. Data are available from the XXX Institutional Data Access / Ethics Committee (contact via XXX) for researchers who meet the criteria for access to confidential data.*<br><br>*The data underlying the results presented in the study are available from (include the name of the third party*	Indicate whether you have had any of the following previous interactions about this manuscript

and contact information or URL).<br>• This text is appropriate if the data are owned by a third party and authors do not have permission to share the data.<br><br>* typeset	
Additional data availability information:	

Click here to access/download;Manuscript;Rice Chlorophyll .pdf

Rice Chlorophyll Inversion Based on UAV Visible Light Imagery

He Jing[1] Wang Bin[2] He Jiachen[1]

[1]School of Geography and Planning, Chengdu University of Technology, Chengdu 610059, China;

[2]Housing and Urban-Rural Development Bureau, Shizhong District, Leshan City, Leshan 614000, Sichuan)

Abstract: As a key substance for crop photosynthesis, chlorophyll content is closely related to crop growth and health. Inversion of chlorophyll content using unmanned aerial vehicle (UAV) visible light images can provide a theoretical basis for crop growth monitoring and health diagnosis. Taking rice at the tasseling stage as the research object, we obtained UAV visible orthophotos of test areas A and B, constructed 14 vegetation indices and 15 texture features, and used the correlation coefficient method to comprehensively analyze the vegetation indices and texture features, and selected four vegetation indices and four texture features for test areas A and B as feature variables to participate in the three types of K-Nearest Neighbors (KNN), decision tree (DT) and AdaBoost models for the chlorophyll content inversion. In the KNN model, the inversion model built with BGRI as the independent variable in region A has the highest accuracy, with R^2 of 0.666 and RSME of 0.79; the inversion model built with RGRI as the independent variable in region B has the

* Funding: This research was supported by the Chengdu City Technology Innovation R&D Project (2022-YF05-01090-SN).

* Corresponding author: He Jing, Ph.D., mainly engaged in research on remote sensing of agriculture from unmanned aerial vehicles, e-mail: xiao00yao@163.com.

highest accuracy, with R^2 of 0.729 and RSME of 0.626. In the decision tree model, the inversion model built with B-variance as the independent variable in region A has the highest accuracy, with R^2 of 0.840 and RSME of 0.464; the inversion model built with G-mean as the independent variable in region B has the highest accuracy, with R^2 of 0.845 and RSME of 0.530. In the AdaBoost model, the inversion model built with R-skewness as the independent variable in region A has the highest accuracy, with R^2 of 0.826 and RSME of 0.642; the inversion model established with g as the independent variable in area B had the highest accuracy, with R^2 of 0.879 and RSME of 0.599. In the comprehensive analysis, the best inversion models in experimental areas A and B were B-variance-decision tree and g-AdaBoost, respectively, and their models were able to carry out the inversion of the chlorophyll content of rice quickly and accurately, which can provide a theoretical basis for the monitoring of the growth and health of the crop.

Keywords: rice; chlorophyll; unmanned aerial vehicle; visible light imagery; vegetation index; textural features.

1 Introduction

Rice is one of the most widely grown food crops worldwide and is important for global food security and economic development. As a key substance for plant photosynthesis, chlorophyll content and distribution directly affect crop growth and photosynthesis. Therefore, quantifying chlorophyll content can indirectly measure nutritional status and is an important indicator for rice health monitoring. Accurate diagnosis of chlorophyll content is of great significance for crop growth monitoring,

nutritional diagnosis, and crop yield estimation, which is an important direction for the development of precision agriculture (Yu et al., 2022; Qin et al., 2016; Moran et al., 2000). Traditional chlorophyll content diagnostic methods mainly include spectrophotometers, fluorescence analysis, and atomic absorption. Although these methods are accurate, they are cumbersome and have many steps, and they can damage crop growth and affect the subsequent growth and development of the crop (Xie et al., 2012; Huang et al., 2007). In response to the shortcomings of traditional chlorophyll measurement methods, instruments have been developed for the rapid, non-destructive determination of the relative chlorophyll content of crops. For example, the SPAD chlorophyll handheld meter in Japan, the Topo chlorophyll content meter in China, and the Zhongke Weiho chlorophyll meter. The chlorophyll meter is mainly used through the 650 nm and 940 nm central band leaf transmittance for the determination of leaf chlorophyll content, and its reading SPAD value can better characterize the leaf greenness (Ai et al., 2000). Many scholars have also proved that SPAD value can effectively replace chlorophyll content through research, such as MARENCO et al. (2009), Uddling et al. (2007), and Li et al. (2014). Although a large number of scholars have proved that the SPAD value can effectively replace chlorophyll content, it cannot be used to assess the chlorophyll content of agricultural crops over a large area due to the limitations of the monitoring area.

In recent years, with the rapid development of remote sensing technology, unmanned aerial vehicle (UAV) remote sensing has been widely used in crop chlorophyll content inversion with its fast and flexible, high spatial resolution and

good timeliness ((Jadan et al., 2020). Cao et al. (2021) aimed at the problem of unclear characteristics of the red edge position in the unmanned aerial hyperspectral inversion of the chlorophyll content of Northeast Japonica rice and determined the position of the red edge of the hyperspectral image of the rice canopy by using six methods, such as linear extrapolation, linear four-point interpolation, etc., which finally showed that linear extrapolation-limit learner and linear extrapolation-logarithmic curve model inversion were more effective. Wang et al. took jujube trees at the fruit-sitting stage as the research object, used UAV visible light images to monitor the SPAD value of jujube tree canopy at the field scale, and constructed univariate regression, multivariate stepwise regression, and random forest regression models for estimating the SPAD value of jujube tree canopy with preferred vegetation indices based on the correlation between 14 types of vegetation indices and the measured SPAD value of jujube trees, to explore the feasibility of estimating the SPAD value of jujube tree canopy using UAV visible light The feasibility of UAV visible light remote sensing images for estimating the SPAD value of date palm canopy was explored. Qiao et al. (2022) used a UAV carrying a multispectral sensor to collect visible and near-infrared images of maize canopy at the jointing stage under six fertilizer application levels to obtain the simple vegetation index, modified vegetation index, and functional vegetation index and established a model for detecting the chlorophyll content of maize canopies by using the partial least squares regression and random forest algorithms.

Visible light imagery has the advantages of low cost, high spatial resolution, and

simple data processing, which further reduces the need for wavelength-remote sensing imagery, thus greatly reducing the difficulty of image acquisition (Xu et al., 2019). The spectral features of UAV visible remote sensing images reflect the visual characteristics of the images, and different features have unique spectral features, and the stability of the spectral features of the images is very good, but they have less spectral information and lack depth information((Wu et al., 2022). With the continuous progress of UAV visible remote sensing imaging technology, the spatial and spectral resolution of feature images has been significantly improved, enabling more and more feature details to be clearly presented(Li et al., 2024). For this reason, after acquiring spectral features, we need to use texture features to describe more comprehensive and accurate feature information to improve the accuracy of target detection (Pan et al., 2022). Qu et al. (2022) and Liu (2019) used UAV visible light images to extract the best variables from the constructed vegetation index to participate in the inversion model operation, which effectively realized the inversion of crop chlorophyll content. However, they neglected the rich texture features of visible light images. Therefore, in this paper, we will extract the spectral information and texture features of visible-light images, compare the inversion results of the two in three different models, namely, K-Nearest Neighbors (KNN), Decision Tree, and AdaBoost, and select the optimal inversion model so as to provide a theoretical basis for the inversion of the chlorophyll content of rice during the tasseling period by visible light images from UAVs.

2 Materials and methods

2.1 Data acquisition

The test area was located in the rice production area of Yao Du Town, Qingbaijiang District, Chengdu City, Sichuan Province, and the data were collected on August 2, 2021 (Rice is in the tasseling stage, which is a critical period for screening high-quality rice grains, and rice begins to enter the reproductive growth stage after tasseling) from 11:30 to 14:20, and the weather was sunny and cloudless at the time of data collection. The data were collected using a DJI Elf 4 Pro-UAV, flying at an altitude of 100 m with 80% overlap in both heading and lateral directions; 10 positioning plates were deployed in the flight area as image control points; and finally, orthophotos were generated using Pix4D. The location map of the test area is shown in Figure 1. Two experimental areas, A and B, were selected as the actual measurement areas of rice chlorophyll content during the tasseling period of this experiment (see Fig. 1), with experimental area A being the area of rice planted artificially and experimental area B being the area of mechanical planting. Chlorophyll measurement data were obtained using the Japanese SPAD-502 Plus handheld chlorophyll meter, and data were collected randomly at 60 measurement points (120 in total) in areas A and B, respectively. Each point was collected five times at different positions of the fully expanded leaves of rice, and the average value was taken as the chlorophyll value on the point. The measured data in each region were randomly assigned in the ratio of 7.5:2.5 as the modeling set and validation set to participate in the inverse model operation.

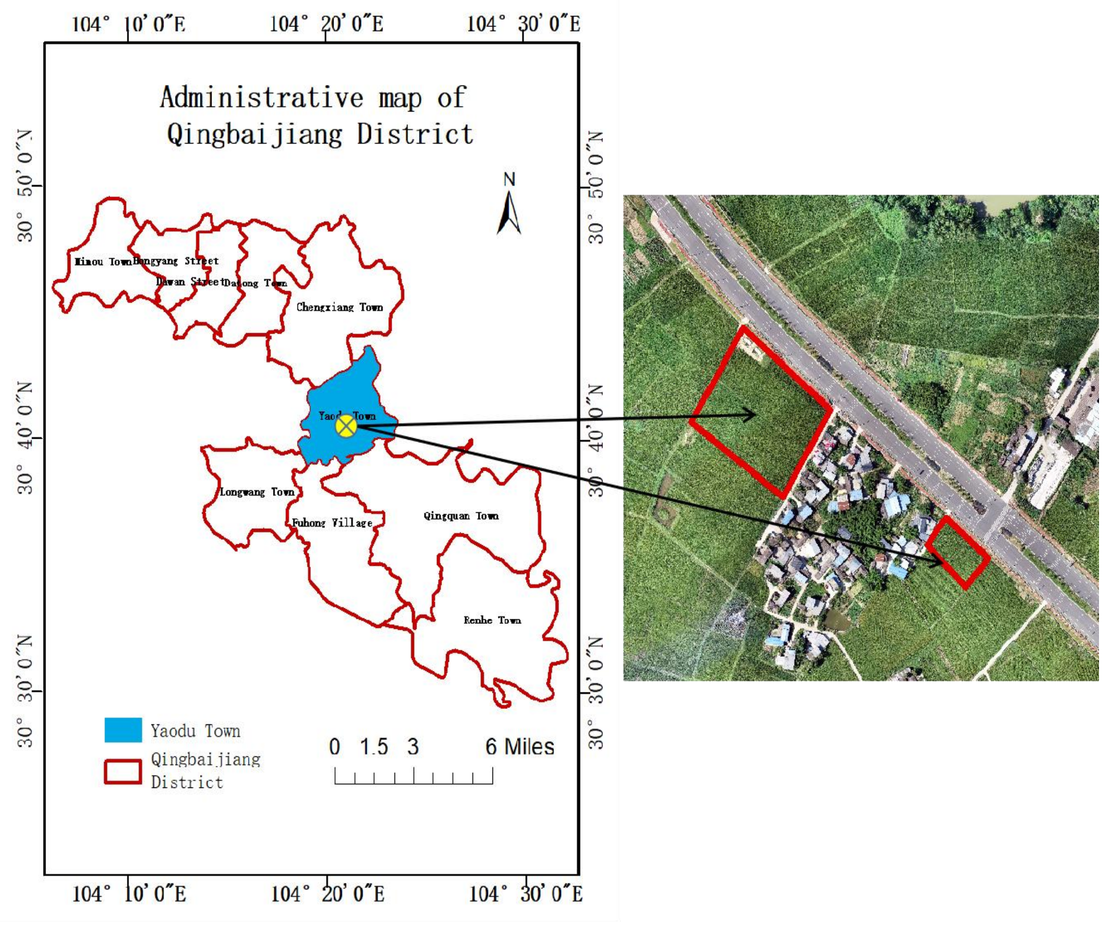

Figure 1 Bitmap of test area

2.2 Feature parameter construction and selection

This experiment uses vegetation indices and texture features as characteristic parameters for the inversion of rice chlorophyll content from UAV visible light images.

The vegetation index is a kind of index reflecting the difference between the reflectance of green vegetation and soil, water, etc. in the visible and near-infrared bands, which is obtained by mathematically combining different bands, and it is a feature used to enhance the information of vegetation (Wang, 2015). Based on previous studies, we constructed a total of 14 visible light vegetation indices, whose formulas and sources are detailed in Table 1.

Table 1 Vegetation index

Vegetation index	formulas	source	Vegetation index	formulas	source
R		*	G-R	G-R	*
G		*	G-B	G-B	*
B		*	GRRI	G/R	Gamon et al., 1999
r	R/（R+G+B）	*	RGRI	R/G	Verrelst et al., 2008
g	G/（R+G+B）	Meng et al., 2020	BGRI	B/G	Sellaro et al., 2010
b	B/（R+G+B）		RBRI	R/B	Wei et al., 2015
R-B	R-B	*	GBRI	G/B	Zhang et al., 2015

Note: 1. R, G and B are the pixel values of the corresponding bands of the visible light image

Texture features are similar shapes containing strong or weak regularity in an image, it is a common visual phenomenon that repeats local structures or rules of arrangement on an image (Gong et al., 2007) and is an important feature of visible light images. Visible light images have three bands, red (R), green (G), and blue (B), and by performing second-order probabilistic statistical filtering on the grey-scale maps of these three bands, five texture features are obtained for each of the three bands: variance, skewness, mean, entropy, and range of values (datarange). Thus we extracted 15 texture features as detailed in Table 2.

Table 2 Texture features

wave band	Texture feature types
R	R-variance、R-skewness、R-mean、R-entropy、R-datarange
G	G-variance、G-skewness、G-mean、G-entropy、G-datarange
B	B-variance、B-skewness、B-mean、B-entropy、B-datarange

In this paper, we used the correlation coefficient method to select the vegetation indices with textural features. The correlation coefficient is a measure of the linear correlation between two variables, and common coefficients used to describe the correlation between variables include Pearson, Spearman, Kendall, Polychoric, Tetrachoric, and Polyserial. Pearson correlation coefficient (Zhang et al., 2019) can numerically measure the correlation between two continuous variables and its results

are directional, so this paper chose to use Pearson correlation coefficient. The range of its value is between -1 and 1. The larger the absolute value, the better the correlation, when $P<0.01$ indicates a highly significant correlation, and $P<0.05$ indicates a significant correlation (Bang et al., 2021).

2.3 Inversion Modelling

Machine learning models are highly adaptable and can handle a variety of complex and variable data. In the inversion of chlorophyll content in rice, machine learning algorithms can accurately estimate the chlorophyll level of rice and provide accurate guidance for agricultural production. Yun et al. (2023) used univariate regression, multivariate regression, and BP neural network algorithm based on principal component analysis to construct the maize SPAD estimation model, screen the optimal model and test it, respectively. Liu et al. (2020) used deep neural networks and convolutional neural networks to establish the relationship model between color information of peanut leaf digital image and leaf chlorophyll content, which provides a theoretical basis for the rapid diagnosis of peanut fertilizer status in production. In this paper, we use three regression models, KNN, Decision Trees and AdaBoost.

KNN is a basic machine learning algorithm that performs classification or regression by measuring the distance between different data points. Its operation procedure is to calculate the distance between the sample to be tested and other known samples, then sort the calculated distances, select the k samples with the smallest distance from the sample to be tested, calculate the average value of the attributes of the k samples and assign it to the sample to be tested (Jiang, 2020). The

KNN algorithm has the advantages of being easy to understand, easy to implement, and there is no specific restriction on the selection of features, and it can deal with a variety of types of data.

Decision tree is a typical method of data feature classification and regression analysis, the results of which are intuitive and easy to understand, the tree structure demonstrates the decision-making process without the need for complex pre-processing or conversion of data. It consists of multiple nodes and edges, and there are two types of nodes: internal nodes and leaf nodes, where the internal nodes represent a feature or attribute of a variable, and the leaf nodes represent a class, while the leaf nodes at the end of the decision tree represent the final decision results, and the other nodes correspond to the judgment rules of the attributes (Wang, 2021). Decision trees have a simple structure, can be constructed with a small amount of data to build a model, and are suitable for regression prediction with small sample data sets.

AdaBoost is an integrated learning algorithm that builds on the framework of the Boosting algorithm by iteratively training multiple weak classifiers and adjusting the weights of the samples according to the performance of each classifier, to gradually construct a strong classifier. During the training process, the weight of misclassified samples will gradually increase, enabling the algorithm to pay more attention to these difficult-to-classify samples. The final strong classifier is a weighted combination of multiple weak classifiers, where the weight of each weak classifier depends on its classification performance on the training set. The use of the AdaBoost algorithm can

effectively avoid overfitting, and the algorithm has a strong learning ability and is not easy to over-adaptation phenomenon (Meng et al., 2022; You et al., 2020; Merler et al., 2007).

2.4 Evaluation indicators

We use the coefficient of determination (R^2) and the root mean square error (RMSE) to judge the reliability and accuracy of the model. Where a larger value of R^2 indicates a better fitting accuracy of the model inversion results, and a smaller value of RSME indicates a higher predictive accuracy of the model (Xie, 2021).

3 Results and Analyses

3.1 Data analysis

The distribution of weeds around the paddy field can be clearly seen from the UAV orthophoto image in Figure 2 (the part drawn by the red line). However, it is difficult to visually identify the weeds in the paddy field through the images. Therefore, we can invert the chlorophyll content of the experimental area and combine it with the chlorophyll results of the weed area to analyze whether there are a large number of weeds in the paddy field and prepare for the subsequent weed control work.

Chlorophyll data analysis: Chlorophyll values measured for rice at the tasseling stage within the experimental areas A and B were statistically analyzed and are shown in Table 3. From Table 3, it can be seen that the measured chlorophyll values ranged from 37.62-43.32 in Region A and 36.53-45.18 in Region B, indicating that the growth in Region A was more homogeneous than that in Region B. In terms of

variance and standard deviation, rice chlorophyll values were less discrete in Region A than in Region B, with less variation in rice growth.

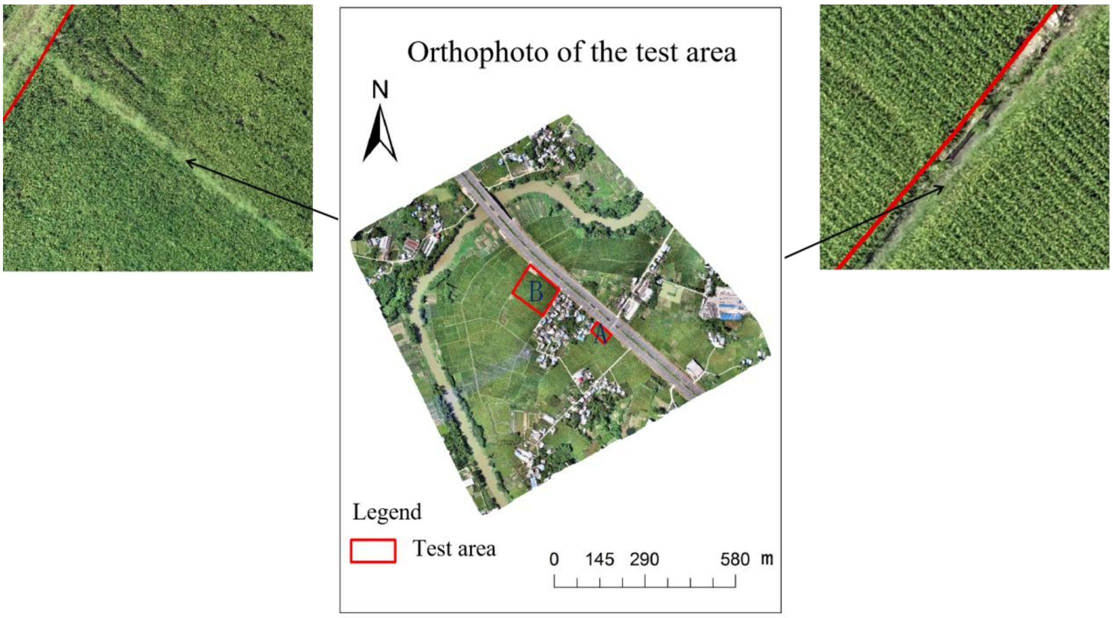

Figure 2 Orthophoto of the test area

Table 3 Orthophoto of the test area

Test area	Number of samples	Minimum value	Maximum value	Mean value	Variance	Standard deviation
A	60	37.62	43.32	39.91	1.47	1.21
B	60	36.53	45.18	41.48	3.92	1.98

3.2 Feature Selection

The correlation analysis of vegetation index, texture characteristics and chlorophyll measured values in areas A and B is shown in Figure 3, which shows the absolute value of the correlation coefficient. As can be seen from Figure 3a, the absolute value of the correlation coefficient between most vegetation indices and chlorophyll content in experimental area A is greater than 0.522. Combining the results of the original correlation analysis, four parameters, g, RGRI, BGRI and GBRI, were selected as characteristic variables, of which g and GBRI were positively and significantly correlated with correlation coefficients of 0.755 and 0.721, respectively; RGRI and BGRI were negatively and highly significantly correlated with correlation

coefficients of -0.746 and -0.718, respectively. Most of the correlation coefficients between vegetation indices and chlorophyll content in experimental area B were greater than 0.42 in absolute value, and four parameters, g, G-R, RGRI and B, were selected as characteristic variables, of which RGRI was positively and significantly correlated, with a correlation coefficient of 0.58, while three, g, G-R and B, were negatively and significantly correlated, with correlation coefficients of -0.74, -0.6 and -0.55, respectively. Combining the correlation of vegetation indices in the two areas shows that more vegetation indices are negatively correlated with chlorophyll. From Fig. 3b, it can be seen that the absolute value of correlation coefficients of most textural features in the test area A is greater than 0.602. Four parameters, R-skewness, G-skewness, B-variance and B-skewness, were selected as feature variables, of which B-variance was positively and significantly correlated with a correlation coefficient of 0.76; R-skewness, G-skewness and B-skewness were negatively and significantly correlated, with a correlation coefficient of 0.76; R- skewness, G-skewness and B-skewness were negatively and significantly correlated, with correlation coefficients of -0.711, -0.739 and -0.78, respectively. The absolute value of correlation coefficients of most texture features in experimental area B was greater than 0.42. Four parameters, R-skewness, G-mean, G-datarange and B-mean, were selected as feature variables, of which R-skewness and G-datarange were positively and significantly correlated, with correlation coefficients of 0.54 and 0.5, respectively; G-mean and B-mean were negatively and significantly correlated, with correlation coefficients of -0.55 and -0.51, respectively. The correlation of texture features in the two areas

shows that more texture features are negatively correlated with chlorophyll. Combining the above analyses, the correlation between vegetation index, texture features and chlorophyll in test area A was better than that in test area B, and in general there were more negative than positive significant correlations.

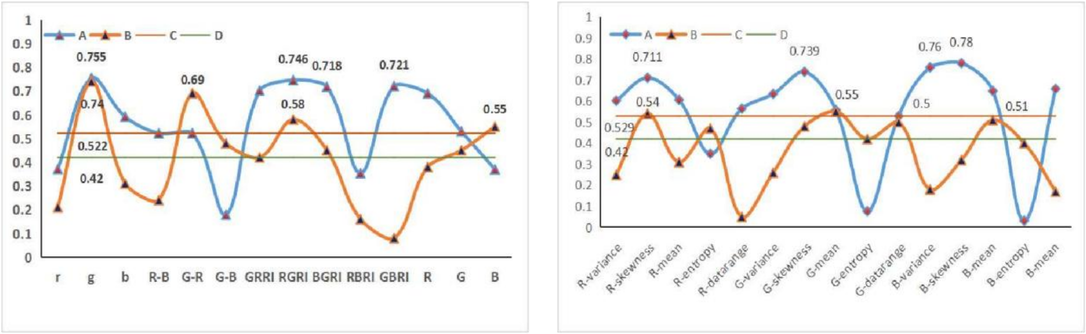

a Absolute value of vegetation index correlation coefficient   b Absolute value of texture feature correlation coefficient

Note: 1. A is the absolute coefficient of regional correlation of a, B is the absolute coefficient of regional correlation of B, C and D are the datum lines of a and B respectively, and their values are determined by extremely significant correlation; 2. * * indicates a very significant correlation

Fig. 3Absolute value diagram of correlation coefficient

3.3 Inversion modelling

Vegetation indices and texture characteristic factors selected from experimental areas A and B were input into KNN, decision tree and AdaBoost models as independent variables, respectively, to establish the chlorophyll inversion model, and the three models with the best results were selected from the inversion results to participate in the subsequent comparison.

3.3.1 K-Nearest Neighbors inversion model

The results of the KNN inversion model are shown in Table 4, which shows that the inversion results of test area B are better than that of test area A. Moreover, the vegetation index, which is involved as an independent variable in the modeling of the two areas, obtains better inversion results than the texture features. The highest

accuracy of both the modelling and validation sets of the inversion model for test area A was the inversion model built with BGRI as the independent variable, with R^2 of 0.583 and RSME of 0.720 for the modeling set, and R^2 of 0.666 and RSME of 0.79 for the validation set. The highest accuracy of both the modeling and validation sets of the B inversion model in the test area was for the inversion model built with RGRI as the independent variable, with R^2 of 0.774 for the modeling set and RSME of 0.760 for the validation set, and R^2 of 0.729 for the validation set and RSME of 0.626 for the validation set.

Table 4 KNN inversion model results

test area	independent variable	modeling set		validation set	
		R^2	RSME	R^2	RSME
A	BGRI	0.583	0.720	0.666	0.790
	GBRI	0.553	0.810	0.640	0.790
	R-skewness	0.425	0.845	0.651	0.826
B	RGRI	0.774	0.760	0.729	0.626
	G-datarange	0.575	1.393	0.624	0.926
	g	0.502	1.349	0.368	1.200

3.3.2 Decision Tree inversion model

The results of the decision tree inversion model are shown in Table 5, which shows that the decision tree inversion model established with the vegetation index and texture features as independent variables achieved better inversion results in both A and B areas, and the inversion accuracies were similar in the two experimental areas of A and B. In both regions, the best inversion results were achieved by participating in the decision tree inversion model with texture features as independent variables, indicating that better results can be achieved by using texture features as independent variables for rice chlorophyll inversion with the decision tree as the inversion model. The highest accuracy in both modeling and validation sets of the inversion models in

the test area A was the inversion model built with B-variance as the independent variable, with R^2 of 0.884 and RSME of 0.341 for the modeling set, and R^2 of 0.840 and RSME of 0.464 for the validation set. The highest accuracy of the modeling set of the B inversion model in the test area is the inversion model constructed with RGRI as the independent variable, with a modeling set R^2 of 0.846 and RSME of 0.254; the highest accuracy of the validation set is the inversion model built with G-mean as the independent variable, with a validation set R^2 of 0.845 and RSME of 0.53.

Table 5 Decision tree inversion model results

test area	independent variable	modeling set		validation set	
		R^2	RSME	R^2	RSME
A	B-variance	0.884	0.341	0.840	0.464
	G-skewness	0.698	0.631	0.790	0.582
	RGRI	0.801	0.497	0.780	0.655
B	G-mean	0.832	0.876	0.845	0.530
	RGRI	0.846	0.254	0.788	0.613
	R-skewness	0.668	1.231	0.775	0.715

3.3.3 AdaBoost inversion model

The results of the AdaBoost inversion model are shown in Table 6, which shows that the inversion model built with the vegetation index and texture features as independent variables achieved better inversion results in both A and B regions. In both areas, the best inversion results were achieved by participating in the AdaBoost inversion model with texture features as independent variables. The results showed that the inversion of rice chlorophyll using AdaBoost as the inversion model with texture features as independent variables could achieve better results. And the inversion result of test area B was better than that of test area A. The highest accuracy of both the modeling and validation sets of the inversion model for test area A was the inversion model built with R-skewness as the independent variable, with R^2 of 0.801

and RSME of 0.497 for the modeling set, and R^2 of 0.826 and RSME of 0.642 for the validation set. The highest accuracy in the modeling set of the B inversion model in the test area is the inversion model constructed with G-mean as the independent variable, with a modeling set R^2 of 0.880 and RSME of 0.477; the highest accuracy in the validation set is the inversion model constructed with g as the independent variable, with a validation set R^2 of 0.879 and RSME of 0.599.

Table 6 AdaBoost inversion model results

test area	independent variable	modeling set		validation set	
		R^2	RSME	R^2	RSME
A	R-skewness	0.801	0.497	0.826	0.642
	B-variance	0.696	0.614	0.805	0.617
	g	0.778	0.525	0.790	0.565
B	g	0.802	0.663	0.879	0.599
	G-datarange	0.730	0.881	0.836	0.565
	G-mean	0.880	0.477	0.819	0.643

3.4 Comparative analysis of inversion models

Figure 4 shows the inversion results with the highest accuracy for the validation set of regression models in test areas A and B. As can be seen from the results of the inversion in test area A in Fig. 4, the best inversion result among the three model optimal inversion results is the B-variance-decision tree model constructed with the texture feature B-variance as the independent variable and the decision tree as the inversion model, and its prediction results and the measured results fit the equation of y=1.1054x-4.2083, and the fitting accuracy R^2 is 0.8402. The best inversion in test area B was the g-AdaBoost model constructed by using the vegetation index g as the independent variable and AdaBoost as the inversion model, with a fitting equation of y=0.8344x+6.6099 for the predicted and measured results, and a fitting accuracy R^2 of 0.8799. Because there are subtle differences in water content and other organic

matter content in soil in different plots, and the data sets collected in different fields have different characteristics, these differences will lead to different performance of models in different data sets, which will affect the selection and performance of models, so the optimal models selected in A and B regions are different. Among the A and B optimal models, the poorer inversion effect is the inversion model constructed with vegetation index and KNN, indicating that the inversion effect of KNN as an inversion model in this visible light rice chlorophyll content inversion has a larger gap than that of the decision tree and AdaBoost models. This is because the effectiveness of the KNN model depends heavily on the number of training samples. Due to the limited number of rice samples, the model may not be able to fully learn the intrinsic patterns and features in the data, resulting in poor performance of the model in predicting the samples. Finally, combining the accuracy of modeling each model with different variables in subsection 2.2, the best inversion model for test area A was selected as a B-variance-decision tree, with a regression equation of y=0.002x+39.466, and the inversion model for test area B was g-AdaBoost, with a regression equation of y=-25.165x+51.922.

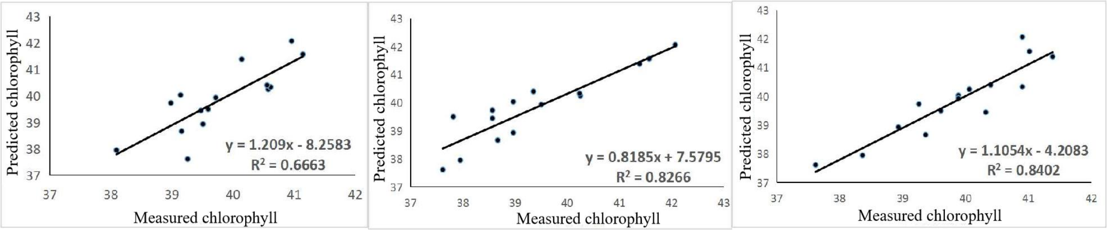

(a) BGRI-KNN (b) R-skewness-AdaBoost (c) B-variance-decision tree

Pilot area A Inversion results for each optimal model

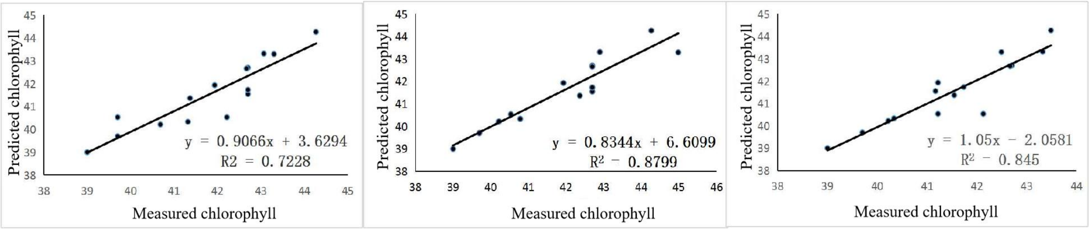

(d) RGRI-KNN (e) g-AdaBoost (f) G-mean-decision tree

Pilot area B Inversion results for each optimal model

Figure 4 Inversion results of optimal model

3.5 Chlorophyll filling and analysis

The regression equations constructed using the best inversion models B-variance-decision tree and g-AdaBoost in experimental areas A and B were used to fill in the chlorophyll maps of rice at the tasseling stage, and the results of filling in the maps are shown in Figure 5. Fig. 5a shows that the chlorophyll values of rice at the tassel stage in experimental area A ranged from 38.5 to 43, with most of them distributed between 40 and 43. In conjunction with Figure 2, it can be seen that the chlorophyll values for the weed areas in the non-rice portion of Area A range from 38.5-40, indicating that within the rice area, a large number of weeds are likely to be present, requiring a manual site visit and targeted weed control. As depicted in Figure 5b, the chlorophyll values for rice during the tapping stage vary within a range of 35-41 within the area. Notably, a significant portion of this area exhibits chlorophyll values predominantly between 35 and 38. A comparative analysis with Figure 2 reveals that there are sections within the area where the chlorophyll levels dip below

35, resembling the chlorophyll concentrations observed in the surrounding weeds. Consequently, it is imperative to conduct a similar field inspection, as done in area A, to assess these areas further. Combining the results of chlorophyll mapping and measured values in the two regions, the predicted chlorophyll results in the test region have a good overlap with the measured results, and the range of values is within the measured data, indicating that the predicted results are reliable. Comparing the two graphs, a and b, the rice is in the same fertility cycle, but there is a large difference in the range of chlorophyll values, indicating that there is a large difference in growth between the two and that the rice grown manually in region A grows significantly better than the rice grown mechanically in region B. Artificial planting is able to adjust the depth and density of planting according to the specific situation and has flexibility under small fields, which is conducive to controlling the growing environment of rice, thus improving the quality of rice to a certain extent. To make the growth of rice in the two regions converge, the nitrogen, phosphorus and potassium contents of rice in regions A and B were compared and analyzed for scientific and precise fertilization, thus promoting the growth of rice in region B without affecting the development of rice in that region in the subsequent growth cycle.

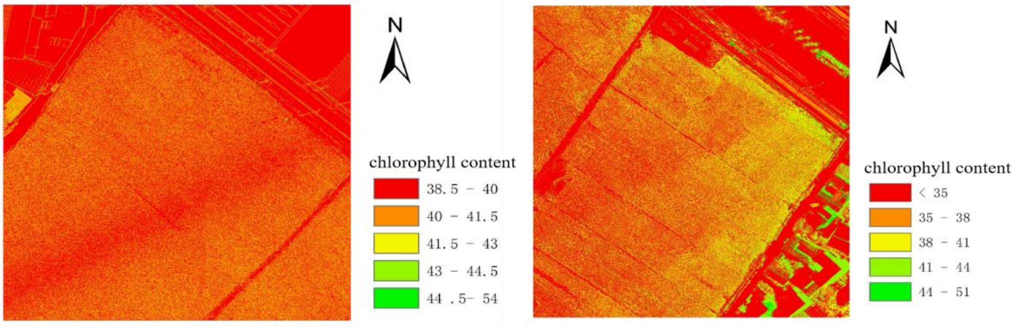

a Pilot area A infill   b Pilot area B infill

Figure 5 Mapping Of Test Area

4 Conclusion and Prospect

4.1 Conclusion

The chlorophyll inversion of rice at the tasselling stage was carried out by UAV visible light data using three inversion models, KNN, decision tree and AdaBoost, and the following conclusions can be drawn from the measured data and inversion results:

(1) By comparing the measured chlorophyll values of the two regions, it can be learned that the rice growth in region A is better and more uniform than that in region B. The measured chlorophyll values are less dispersed, and the final inversion results also show that the rice growth in region A is more uniform than that in region B. The inversion results also show that the rice growth in region A is more uniform than that in region B.

(2) There is a good correlation between vegetation index and texture features extracted using UAV visible light images and chlorophyll. The highest absolute value of correlation coefficient for vegetation index g is 0.805 in region A; the highest absolute value of correlation coefficient for texture feature B-skewness is 0.83, and the overall correlation of texture features is higher compared to the vegetation index. In Region B as a whole, the correlation coefficients of vegetation index and Region B, as a whole, have lower absolute values of correlation coefficients for vegetation indices and texture features, with the highest absolute value of correlation coefficient for vegetation index g at 0.74, and the highest absolute value of correlation coefficient for texture feature G-mean at 0.55. As a whole, the correlation between vegetation indices and texture features in Region A is better than that in Region B.

(3) The inversion models constructed based on vegetation indices and texture features with K-neighbourhood (KNN), Decision Tree and AdaBoost, respectively, all achieved better inversion results, and Decision Tree and AdaBoost achieved the best results in the inversion of rice chlorophyll during the tasseling stage in regions A and B, respectively. The best inversion model in region A was identified as a B-variance-decision tree, with a fitting equation of y=1.1054x-4.2083, a fitting accuracy of R^2 of 0.8402, and an RSME of 0.464; and the best inversion model in region B was identified as g-AdaBoost, with a fitting equation of y=0.8344x+6.6099, and an accuracy of R^2 of 0.87%, and an RSME of 0.8402. accuracy R^2 was 0.8799 and RSME was 0.599.

(4) The best inversion model for regions A and B was used to fill in the map. The results of filling in the map show that the range of chlorophyll values in regions A and B overlapped with the range of measured data, indicating that the inversion results are somewhat reliable. Further analysis of the data shows that the growth of rice in region A is more uniform and better than that in region B. It is possible to analyze and compare the rice nitrogen, phosphorus, potassium, and other trace elements in regions A and B, and then carry out targeted fertilization in region B to promote the growth of rice in this region. At the same time can be filled through the chlorophyll map of the rice within the weed identification, and then to achieve accurate dosing and weeding.

4.2 Prospect

Restricted by objective factors such as research conditions and experimental time,

there are still improvements in this study, which need to be explored and improved more deeply in future scientific research, as follows:

(1) One of the notable problems with experiments in doing data analysis or machine learning is model selection and transferability. The current study only considered a limited number of models and datasets, leading to differences in model selection in regions (A and B) that are geographically similar and have little environmental differences. Therefore, future research elements should explore more types of models, collect more datasets from other life cycles of rice, and improve the migratory nature of models.

(2) In this experimental study area, the two fields A and B exhibit substantial variations in both area size and planting methodologies, coupled with nuanced differences in soil organic matter content. Subsequent experiments can be carried out to construct soil nutrient content assessment models by selecting similar experimental field samples in terms of soil fertility and fertilizer application, making the constructed inversion models of practical production significance.

Abbreviations

UAV: Unmanned aerial vehicle; KNN: K-Nearest Neighbors; R^2: coefficient of determination; RMSE: Root mean square error.

Disclosure statement

The authors declare that they have no known competing financial interests or personal relationships that could have appeared to influence the work reported in this paper.

Funding

This study was supported by the Chengdu City Technology Innovation R&D Project (2022-YF05-01090-SN).

Notes on contributor(s)

He Jing: Conceptualization, Methodology, Writing - review & editing; **Wang Bin**: Data curation, Writing - original draft; **He Jiachen**: Investigation and Translate.

Data Availability

The datasets generated during and/or analyzed during the current study are available from the corresponding author on reasonable request.

References:

Yu Yue, Yu Haiye, Li Xiaokai, Wang Hongjian, Liu Shuang, Zhang Lei, Sui Yuanyuan. 2022. Hyperspectral inversion model for SPAD of rice leaves based on optimized specral index. Spectroscopy and Spectral Analysis. 42(04):1092-1097.

Qin Zhanfei, Chang Qingrui, Shin Jian, Yu Yang, Liu Jiaqi. 2016. Red Edge Characteristics andSPAD Estimation Model Using Hyperspectral Data for Rice in Ningxia Irrigation Zone[J]. Journal of Wuhan University (Information Science Edition). 41(09):1168-1175.

Moran J A , Mitchell A K , Goodmanson G , et al. 2000. Differentiation among effects of nitrogen fertilization treatments on conifer seedlings by foliar reflectance: a comparison of methods[J]. Tree Physiology. 20(16):1113-1120.

Xie Chuanqi, He Yong, Li Xiaoli, Liu Fei, Du Pengpeng. 2012. Study of Detection of SPAD Value in Tomato Stressed by Grey Mold Based on Hyperspectral Technique[J]. Spectroscopy and Spectral Analysis. 32(12):3324-3328.

Huang Chidu, Hu Xiaosong, Liao Xiaojun, Wu Jihong. 2007. The advancement of chlorophyll [J]. China Food Additives. (03):114-118.

Ai Tiancheng, Li Fangmin, Zhou Zhian, Sharla Cheung, Wu Hairong. 2000. Relationship between Chlorophyll Meter Readings(SPAD Readings)and Chlorophyll Content of Crop Leaves [J]. Journal of Hubei Agricultural College. (01):6-8.

Marenco R A, Antezana-Vera S A, Nascimento H C S. 2009. Relationship between specific leaf area, leaf thickness, leaf water content and SPAD-502 readings in six Amazonian tree species[J]. Photosynthetica. 47(2): 184-190.

Uddling J, Gelang-Alfredsson J, Piikki K, et al. 2007. Evaluating the relationship between leaf chlorophyll concentration and SPAD-502 chlorophyll meter readings[J]. Photosynthesis Research. 91(1):37-46.

Li Xuhua, Hu Qiang, Pan Yihong, Zhang Xiaolong, Wang Juan. 2014. Correlation analysis of chlorophyll content and SPAD values in flue-cured tobacco of different maturity[J]. Henan Agricultural Sciences. 43(03):47-52+58.

Jia Dan, Chen Pengfei. 2020. Effect of Low-altitude UAV Image Resolution on Inversion of Winter Wheat Nitrogen Concentration[J]. Journal of Agricultural Machinery. 51(07):164-169.

Cao Yingli, Jiang Kailun, Liu Yadi, Zheng Zhengxin, Xiao Wen, Yu Fenghua. 2021. Rice Chlorophyll Inversion based on Hyperspectral Red Edge Retrieval[J]. Journal of Shenyang Agricultural University. 52(06):718-728.

Wang Yongdong, Nigra Tursun, Zheng Jianghua, et al. 2024. Preliminary estimation of canopy SPAD value of jujube tree during fruit-setting period based on UAV visible image[J]. Jiangsu Agricultural Sciences. 52(06):206-215.

Qiao L, Tang W, Gao D H, Zhao R M, An L L, Li M Z, Sun H, Song D. 2022. UAV-based chlorophyll content estimation by evaluating vegetation index responses under different crop coverages[J]. Computers and Electronics in Agriculture, 196, Article 106775.

Xu WC; Lan YB; Li YH; Luo YF; He ZY. 2019. Classification method of cultivated land based on UAV visible light remote sensing. International Journal of Agricultural and Biological Engineering. Vol. 12: 103-109.

Wu Fang, Li Yu, Jin Dingjian, et al. 2022. Application of 3D information extraction technology of ground obstacles in the flight trajectory planning of UAV airborne geophysical exploration[J]. Remote sensing of natural resources. 34(01):286-292.

Li Hua, Li Guo. 2024. Research on ground object extraction technology for visible light remote sensing images of UAVs[J]. Computer Measurement and Control. 32(02):250-255.

Pan Gaofeng, Fan Yuan, Ru Yu, et al. 2022. Low-texture monocular visual simultaneous localization and mapping algorithm based on point-line feature fusion[J]. Computer Application. 42(7):2170-2176.

Qu Yiling, Tang Yan, Zhou Zhongsheng, Yan Fang, Wang Juzhong, Xu Huaixing, Hu Lulu, Xu Xiaojun. 2022. Study on model simulation of relative chlorophyll content of moso bamboo based on UAV visible light image[J]. Journal of Jiangxi Agricultural University. 44(01):139-150.

Liu Xiaohui. 2019. Inversion of wheat chlorophyll content and yield based on unmanned aerial vehicle images[D]. Anhui University.

Wang Xiaoqin, Wang Miaomiao, Wang Shaoqiang, Wu Yundong. 2015. Extraction of vegetation information from visible unmanned aerial vehicle images[J]. Journal of Agricultural

Engineering. 31(05):152-157+159+158.

Meng Zhuanchao, Zhao Jing, Lan Yubin, Yan Chunyu, Yang Dongjian, Wen Yuting. 2020. SPAD inversion model of corn canopy based on UAV visible light image [J]. Journal of Agricultural Machinery. 51(S2):366-374.

Gamon J A, Surfus J S. 1999. Assessing leaf pigment content and activity with a reflectometer[J]. The New Phytologist. 143(1): 105-117.

Verrelst J, Schaepman M E, Koetz B, et al. 2008. Angular sensitivity analysis of vegetation indices derived from CHRIS/PROBA data[J]. Remote Sensing of Environment. 112(5): 2341-2353.

Sellaro R, Crepy M, Trupkin S A, et al. 2010. Cryptochrome as a sensor of the blue/green ratio of natural radiation in Arabidopsis[J]. Plant Physiology. 154(1): 401-409.

Wei Quanquan, Li Litao, Ren Tao, Wang Zhen, Wang Shaohua, Li Xiaokun, Cong Rihuan, Lu Jianwei. 2015. Diagnosing nitrogen nutrition status of winter rapeseed via digital image processing technique[J]. China Agricultural Science. 48(19):3877-3886.

Zhang Peisong, Sun Yiming, Guo Pengtao, Yuan Zhongzhi, Yang Hongzhu, Bei Meimei, Luo Wei. 2015. Study on predicting nitrogen content of rubber tree leaf by digital image analysis[J]. Acta Tropical Crops. 36(12):2120-2124.

Gong Hongju, Ji Changying. 2007. Estimating yield of wheat spike with its texture features based on image processing technology[J]. Journal of Agricultural Machinery. (12):116-119.

Zhang Aiwu, Dong Zhe, Kang Xiaoyan. 2019. Feature selection algorithm of airborne lidar combined with hyperspectral images based on XGBoost[J]. China Laser. 46(4): 40400.

Ban Songtao, Tian Minglu, Chang Qingrui, Wang Qi, Li Fenling. 2021. Estimation of Rice Leaf Phosphorus Content Using UAV-based Hyperspectral Images[J]. Journal of Agricultural

Machinery. 52(08):163-171.

Yun Binyuan, Zhang Hao, Zhai Yongquan, et al. 2024. Estimation method for canopy SPAD values of maize based on soil nitrogen level[J/OL]. Journal of Ecology: 1-11.

Liu Shiyuan, Liang Jin, Wang Shuaibin, et al. 2020. Research on Monitoring of Peanut Leaf Chlorophyll Content Based on UAV Remote Sensing[J]. Journal of Peanut. 49 (02): 21-27+35. DOI: 10.14001/j.ISSN.1002-4093.2020.00.

Jiang Fugen. 2020. Inversion Study on optimized kNN method of vegetation leaf area index[D]. Central South University of Forestry and Technology.

Wang Ying. 2021. Application of decision tree model in soybean futures price trend forecast[D]. Northwest Normal University.

Meng Xianwei, Tang Jinjun, Wang Zhe. 2022. Trajectory prediction of vehicles based on LSTM-Ada Boost considering lane-change intention[J]. Computer Engineering and Application. 58(13):280-287.

You Wenxia, Zhao Di, Yong-Hua Wu, Huang Yaoyu, Shen Kun, Li Wenwu. 2020. Research on maximum load forecasting of power distribution area based on AdaBoost integrated learning[J]. Journal of China Three Gorges University (Natural Science Edition). 42 (06): 92-96.

Merler, S, Caprile, et al. 2007. Parallelizing AdaBoost by weights dynamics[J]. Computational Statistics and Data Analysis.

Xie Lili. 2021. Rice quality estimation based on multi-platform hyperspectral remote sensing data[D]. Zhejiang University.
