# Supplementary material for: Chlorophyll inversion in rice based on visible light images of different planting methods
Source: PLoS One. 2025 Mar 24;20(3):e0319657. doi: 10.1371/journal.pone.0319657 (PMC11932478; doi:10.1371/journal.pone.0319657)
Supplement: Attachment — Submitted filename: Response to Reviewers.docx [file pone.0319657.s002.docx]

**Dear Editor:**

Manuscript ID **PONE-D-24-27430** entitled "**Spectral information and texture features synergize for rice chlorophyll inversion**" which has been revised.

We are truly grateful to yours critical comments and thoughtful suggestions. Based on these comments and suggestions, **we have made careful modifications on the manuscript, including title, introduction, and discussion.** **Besides, we modified the meets of the manuscript according to PLOS ONE's style requirements.** All changes made to the text are in **red** color.

We hope the new manuscript will meet your magazine’s standard. Please do forward our heartfelt thanks to these experts. We look forward to hearing from you soon for a favorable decision.

**We tried our best to improve the manuscript and made a significant change in the manuscript. Below you will find our point-by-point responses to the reviewers’ and editor’s comments/ questions.**

**Comments from the editors and reviewers:**

The manuscript must be corrected in all points indicated by the reviewers, such as:

1) The author's title does not effectively summarise the work.

**Response:** Thank you very much for the suggestion. We have changed the title to ‘Spectral information and texture features synergize for rice chlorophyll inversion’ to better summarise the work of this paper.

2) The authors do not provide a clear description of the research objectives in the introduction.

**Response:**Thank you very much for the suggestion. We have revised the introduction to add a clear description of the research objectives, as described below: Therefore, in this paper, we extracted the spectral information and texture features of UAV visible images, constructed 14 spectral indices and 15 texture features, and selected the feature variables that were highly correlated with the measured rice chlorophyll content as the input variables of the model using correlation analysis. We compared the inversion results of these two parameters in three different models, namely, K-neighborhood (KNN), Decision Tree (DT), and AdaBoost, and evaluated the performance differences among these three inversion models in predicting the chlorophyll content of rice, and selected the best inversion model. In addition, to explore the applicability of the models in different planting types of farmland, we selected two representative planting types of farmland (manual planting and mechanical planting). We tested the inversion results of the three models in these two types of farmland, to deeply explore the applicability conditions of the models as well as the influence of the planting types of cropland on the inversion results. It provides the theoretical basis and practical guidance for the inversion of chlorophyll content of rice at the tasseling stage by UAV visible light images.

3) The marginal contributions, especially the innovative nature of the manuscript, are not well written.

**Response:** Thank you very much for the suggestion. For the description of the paper's innovative nature, we have revised and summarized it in the introduction section as follows: We compared the inversion results of these two parameters in three different models, namely, K-neighborhood (KNN), Decision Tree (DT), and AdaBoost, and evaluated the performance differences among these three inversion models in predicting the chlorophyll content of rice, and selected the best inversion model. In addition, to explore the applicability of the models in different planting types of farmland, we selected two representative planting types of farmland (manual planting and mechanical planting). We tested the inversion results of the three models in these two types of farmland, to deeply explore the applicability conditions of the models as well as the influence of the planting types of cropland on the inversion results. It provides the theoretical basis and practical guidance for the inversion of chlorophyll content of rice at the tasseling stage by UAV visible light images.

4) The objectives of the study are not clear.

**Response:** Thank you very much for the suggestion. The goal of our research is to invert the chlorophyll content of rice leaves at the tasseling stage using vegetation indices and texture feature parameters combined with various machine learning algorithms, to select the optimal inversion model, and to explore the planting method suitable for Chengdu's farmland, so as to provide a theoretical basis for the inversion of chlorophyll content of rice at the tasseling stage using UAV visible light imagery. This content we have modified in the introduction part.

5) The paper lacks discussion, especially with other similar methods.

**Response:** Thank you very much for the suggestion. We have added a discussion section to explore the three parts of characterization parameters, inversion models and planting methods. And we compared with other similar research methods to highlight the innovation of this paper.

6) The discussion should indicate the advantages and disadvantages of the method, especially applicability, cost, and detection accuracy.

**Response:** Thank you very much for the suggestion. In the first subsection of our discussion, we explored the effect of inversion of the vegetation index with the texture feature parameter and verified the generalization of these two parameters across different cropping patterns. We explore the generalization ability and applicability of the model in the second subsection of the discussion. the AdaBoost model allows the use of different types of weak learners, which provides the model with greater flexibility and adaptability, and significantly improves the prediction performance of the overall model. the inversion effect of KNN as an inversion model has a larger gap than that of the decision tree and the AdaBoost model, which suggests that the model is affected by the non-uniformity of the distribution of the training samples, resulting in biased prediction results. uniformity of the training samples, resulting in biased prediction results. In the third subsection of our discussion, we explore the advantages and disadvantages of manual versus mechanical planting, and analyze planting methods suitable for the farmland of Chengdu.

When submitting your revision, we need you to address these additional requirements.

1. Please ensure that your manuscript meets PLOS ONE's style requirements, including those for file naming. The PLOS ONE style templates can be found at [https://journals.plos.org/plosone/s/file?id=wjVg/PLOSOne_formatting_sample_main_body.pdf](https://journals.plos.org/plosone/s/file?id=wjVg/PLOSOne_formatting_sample_main_body.pdf) and [https://journals.plos.org/plosone/s/file?id=ba62/PLOSOne_formatting_sample_title_authors_affiliations.pdf](https://journals.plos.org/plosone/s/file?id=ba62/PLOSOne_formatting_sample_title_authors_affiliations.pdf)

**Response:** Thank you very much for the suggestion. We have revised the manuscript meets according to the PLOS ONE's style requirements.

2. Please update your submission to use the PLOS LaTeX template. The template and more information on our requirements for LaTeX submissions can be found at [http://journals.plos.org/plosone/s/latex.](http://journals.plos.org/plosone/s/latex.)

**Response:** Thank you very much for the suggestion. We have revised the manuscript meets according to the PLOS ONE's style requirements.

3. In your Methods section, please provide additional information regarding the permits you obtained for the work. Please ensure you have included the full name of the authority that approved the field site access and, if no permits were required, a brief statement explaining why.

**Response:** Thank you very much for the suggestion. We were not required to provide permits for our work because the study area we visited was part of our contracted test plots.

4. Please note that PLOS ONE has specific guidelines on code sharing for submissions in which author-generated code underpins the findings in the manuscript. In these cases, we expect all author-generated code to be made available without restrictions upon publication of the work. Please review our guidelines at [https://journals.plos.org/plosone/s/materials-and-software-sharing#loc-sharing-code](https://journals.plos.org/plosone/s/materials-and-software-sharing#loc-sharing-code) and ensure that your code is shared in a way that follows best practice and facilitates reproducibility and reuse.

**Response:** Thank you very much for the suggestion. We code is shared in a way that follows best practice and facilitates reproducibility and reuse.

5. Thank you for stating the following financial disclosure: "Chengdu City Technology Innovation R&D Project (2022-YF05-01090-SN)"  Please state what role the funders took in the study.  If the funders had no role, please state: "The funders had no role in study design, data collection and analysis, decision to publish, or preparation of the manuscript." "If this statement is not correct you must amend it as needed. Please include this amended Role of Funder statement in your cover letter; we will change the online submission formon your behalf.

**Response:** Thank you very much for the suggestion. The funders had no role in study design, data collection and analysis, decision to publish, or preparation of the manuscript. And we have deleted that sentence.

6. We note that your Data Availability Statement is currently as follows: Indicate whether you have had any of the following previous interactions about this manuscript. Please confirm at this time whether or not your submission contains all raw data required to replicate the results of your study. Authors must share the “minimal data set” for their submission. PLOS defines the minimal data set to consist of the data required to replicate all study findings reported in the article, as well as related metadata and methods ([https://journals.plos.org/plosone/s/data-availability#loc-minimal-data-set-definition](https://journals.plos.org/plosone/s/data-availability#loc-minimal-data-set-definition)).

**Response:** Thank you very much for the suggestion. Our submission contains all the raw data needed to replicate the results of the study. Our research data is original and we have removed this statement.

7. Please amend the manuscript submission data (via Edit Submission) to include author Dr. Wang Bin and Dr. He Jiachen.

**Response:** Thank you very much for the suggestion. We will amend the manuscript submission data (via Edit Submission) to include author Dr. Wang Bin and Dr. He Jiachen.

8. We note that Figures 1 and 2 in your submission contain [map/satellite] images which may be copyrighted. All PLOS content is published under the Creative Commons Attribution License (CC BY 4.0), which means that the manuscript, images, and Supporting Information files will be freely available online, and any third party is permitted to access, download, copy, distribute, and use these materials in any way, even commercially, with proper attribution. For these reasons, we cannot publish previously copyrighted maps or satellite images created using proprietary data, such as Google software (Google Maps, Street View, and Earth). For more information, see our copyright guidelines: [http://journals.plos.org/plosone/s/licenses-and-copyright.](http://journals.plos.org/plosone/s/licenses-and-copyright.)

**Response:** Thank you very much for the suggestion. The Figures 1 and 2 of the paper are visible light images taken by us using a drone and do not contain copyrighted images.

**Second Comments from the editors：**

5. We note that you state the following regarding your Figures: "Thank you very much for the suggestion. The Figures 1 and 2 of the paper are visible light images taken by us using a drone and do not contain copyrighted images."

We note that your Figure 1 contains a map image. At this time, please kindly clarify the following regarding Figure 1:

a) Where did the authors obtain the map image in Figure 1?

b) Please state whether the map image has been previously copyrighted to your knowledge.

c) If the map image has been previously copyrighted, we require specific consent from the copyright holder to publish these images in PLOS ONE, under the CC BY 4.0 license. To seek permission from the copyright owner to publish your map figures under the specific Creative Commons Attribution License (CCAL), CC BY 4.0, please contact them with the following text and PLOS ONE Request for Permission form (https://journals.plos.org/plosone/s/file?id=c4ff/Content%20Copyright%20Permission%20Form.pdf):

“I request permission for the open-access journal PLOS ONE to publish XXX under the Creative Commons Attribution License (CCAL) CC BY 4.0 (http://creativecommons.org/licenses/by/4.0/). Please be aware that this license allows unrestricted use and distribution, even commercially, by third parties. Please reply and provide explicit written permission to publish XXX under a CC BY license.”

**Response:** Thank you very much for the suggestion. We have removed the location map of the study area in Figure 1 because it involves copyrighted map imagery and that the location map has no impact on the experiment.
